# High Adherence to Antimalarials and Antibiotics under Integrated Community Case Management of Illness in Children Less than Five Years in Eastern Uganda

**DOI:** 10.1371/journal.pone.0060481

**Published:** 2013-03-29

**Authors:** Joan N. Kalyango, Elizeus Rutebemberwa, Charles Karamagi, Edison Mworozi, Sarah Ssali, Tobias Alfven, Stefan Peterson

**Affiliations:** 1 Department of Public Health Sciences, Global Health, Karolinska Institutet, Stockholm, Sweden; 2 Clinical Epidemiology Unit, Makerere University College of Health Sciences, Kampala, Uganda; 3 Department of Pharmacy, Makerere University College of Health Sciences, Kampala, Uganda; 4 Department of Health Policy, Planning and Management, School of Public Health, Makerere University College of Health Sciences, Kampala, Uganda; 5 Department of Paediatrics and Child Health, Makerere University College of Health Sciences, Kampala, Uganda; 6 Department of Gender and Women Studies, Makerere University, Kampala, Uganda; 7 Department of Paediatrics, Sach's Children's Hospital, Stockholm, Sweden; 8 International Maternal and Child Health Unit, Uppsala University, Uppsala, Sweden; Kenya Medical Research Institute - Wellcome Trust Research Programme, Kenya

## Abstract

**Background:**

Development of resistance to first line antimalarials led to recommendation of artemisinin based combination therapies (ACTs). High adherence to ACTs provided by community health workers (CHWs) gave reassurance that community based interventions did not increase the risk of drug resistance. Integrated community case management of illnesses (ICCM) is now recommended through which children will access both antibiotics and antimalarials from CHWs. Increased number of medicines has been shown to lower adherence.

**Objective:**

To compare adherence to antimalarials alone versus antimalarials combined with antibiotics under ICCM in children less than five years.

**Methods:**

A cohort study was nested within a cluster randomized trial that had CHWs treating children less than five years with antimalarials and antibiotics (intervention areas) and CHWs treating children with antimalarials only (control areas). Children were consecutively sampled from the CHWs' registers in the control areas (667 children); and intervention areas (323 taking antimalarials only and 266 taking antimalarials plus antibiotics). The sampled children were visited at home on day one and four of treatment seeking. Adherence was assessed using self reports and pill counts.

**Results:**

Adherence in the intervention arm to antimalarials alone and antimalarials plus antibiotics arm was similar (mean 99% in both groups) but higher than adherence in the control arm (antimalarials only) (mean 96%). Forgetfulness (38%) was the most cited reason for non-adherence. At adjusted analysis: absence of fever (OR = 3.3, 95%CI = 1.6–6.9), seeking care after two or more days (OR = 2.2, 95%CI = 1.3–3.7), not understanding instructions given (OR = 24.5, 95%CI = 2.7–224.5), vomiting (OR = 2.6, 95%CI = 1.2–5.5), and caregivers' perception that the child's illness was not severe (OR = 2.0, 95%CI = 1.1–3.8) were associated with non-adherence.

**Conclusions:**

Addition of antibiotics to antimalarials did not lower adherence. However, caregivers should be adequately counseled to understand the dosing regimens; continue with medicines even when the child seems to improve; and re-administer doses that have been vomited.

## Introduction

Following widespread drug resistance to various mono therapies for the treatment of malaria including chloroquine, sulphadoxine-pyrimethamine (SP), or amodiaquine, the World Health Organization (WHO) recommended use of combination therapies in 2001 [1]. Uganda initially adopted a combination of chloroquine and SP as the first line drugs for uncomplicated malaria. However, resistance levels to this combination rose rapidly, necessitating further changes in malaria policy in 2005, and use of artemisinin based combination therapies (ACTs) was recommended [2]. Uganda along with some other countries adopted artemether-lumefantrine (AL) as the first line treatment for uncomplicated malaria [3].

Unlike chloroquine which required once-a-day dosing over a period of three days and SP which was a single dose, AL has to be taken twice daily over three days resulting in more frequent administration than for chloroquine and SP. In addition, the amount of AL to be taken per dose is higher than that for chloroquine. For example, a three year old child who previously took one tablet of chloroquine has to take two tablets of AL. Increased number of pills and frequency of administration of medicines have been associated with poor adherence [4] and this creates concern of possible poor adherence with AL. Poor adherence to medicines increases the risk of drug resistance to medicines as well as poor treatment outcomes [5]. The negative impact of these problems are likely to be felt mostly among the populations that bear the biggest burden of malaria i.e. children under five years [6], further worsening morbidity and mortality in this age-group.

Worldwide, several efforts have been made over the years to reduce mortality in children less than five years including the *home management of fever strategy* that was adopted in Uganda in 2002 [7]. This strategy involved treatment of children with fever at home by community health workers (CHWs)[8], a cadre of informal health workers with no basic medical training [9] but who were trained on malaria management for one week. The CHWs initially treated children using Homapak®, a combination of chloroquine and SP but the antimalarial policy change in 2005 also necessitated a change in medicines used for home management of fever to AL. Several studies were conducted in Uganda and other countries to evaluate the feasibility of CHWs using ACTs and the findings from these studies favored rolling out ACTs to CHWs [10], [11], [12].

In 2010, the *integrated community case management of childhood illnesses strategy* (ICCM) which involves integrated management of children with multiple illnesses in the community was adopted [13] following recommendations by WHO [14]. The recommendations for integrated management of illnesses in children resulted from findings that many children suffered from multiple illnesses or illnesses with symptoms suggestive of different diseases [15], [16], [17]. Under the ICCM strategy, children can receive antibiotics, oral rehydration salts (ORS) and antimalarials from CHWs so that they promptly receive appropriate treatment for pneumonia, diarrhea and malaria; the most common causes of mortality for children under 5 years of age. It is important that children adhere to the treatment in order to get good effects from the medicine and to prevent the development of resistance, especially with the more widespread use that is expected with community management of illnesses. Studies among children treated by CHWs using ACTs alone have found levels of adherence of 73–97% [12], [18]. We hypothesized that the increased pill burden due to addition of antibiotics to antimalarials, together with the more complicated medicines instructions, could reduce adherence. This study therefore aimed at comparing adherence to antimalarials alone versus antimalarials combined with antibiotics under ICCM in children less than five years.

## Methods

### Ethics statement

Approval to conduct the study was given by the School of Public Health Higher Degrees Research and Ethics Committee (reference IRB00005876), the Uganda National Council of Science and Technology (reference HS 898), the administration of Iganga-Mayuge Health and Demographic Surveillance Site (HDSS) and the local administration of the villages from where the children were enrolled. Caregivers of the children were informed about the study and then written informed consent was obtained from caregivers that allowed their children to be enrolled into the study. Two copies of the informed consent documents were signed by each caregiver and the person conducting the informed consent. One of the copies of the signed consent document was taken by the caregiver and the other was retained in our records. Access to the data was restricted to the investigators.

### Study design and setting

A cohort study was nested within a cluster randomized trial in Iganga-Mayuge HDSS in October to November 2011. The cluster randomized trial was comparing under-five mortality in areas where CHWs treated children with fever using antimalarials only **(control areas)** and those where CHWs treated children with fever or respiratory symptoms or both using antimalarials or antibiotics or a combination of both as appropriate **(intervention areas)** (Trial Registration number: ISRCTN52966230). The CHWs in both the intervention and control areas treat children with antimalarials or antibiotics or refer them following symptom based illness classification made according to the WHO integrated management of childhood illness (IMCI) guidelines [19]. Children are treated with antimalarials if they present with fever or a history of fever within the previous 24 hours with no danger signs (convulsions, repeated vomiting, failure to feed, lethargy/unconsciousness, chest in-drawings, noisy breathing, severe dehydration, and pallor). In addition, the CHWs in the intervention arm treat children using antibiotics if they have non-severe respiratory symptoms (cough and difficult breathing or fast breathing (≥50 breaths per minute in children aged four to 12 months and ≥40 breaths per minute in children 12 to 59 months)). Children with danger signs in both arms are referred to nearby health facilities. The CHWs do not use rapid diagnostic tests (RDTs) because the trial commenced before WHO made the recommendation of universal parasite based diagnosis RDTs in 2010 [20]. The trial has been described elsewhere [21], [22]. Iganga-Mayuge HDSS where the study was conducted is mainly rural with only 10% of the population living in peri-urban areas. It has a population of about 70000 and of these about 11000 are children less than five years.

#### Treatment regimens given by CHWs in Iganga-Mayuge HDSS

Children receive age-based doses of pre-packaged medicines in blister packs with different colors of the packs for easy identification. The children aged 4–35 months receive one tablet of artemether-lumefantrine (20 mg artemether, 120 mg lumefantrine) twice daily for three days and the medicine is contained in a yellow pack. Children aged 36–59 months receive two tablets of artemether-lumefantrine twice daily for three days contained in a blue pack. The children in the intervention arm can also receive 3-day courses of amoxicillin (125 mg) as follows: one tablet twice daily for children aged 4–11 months (pink pack), two tablets twice daily for children 12–35 months (green pack) and three tablets twice daily for children 36–59 months (red pack). The artemether-lumefantrine blister packs contain illustrations to ease understanding and recall of medicines administration instructions.

### Study participants

Children aged 4–59 months that had been treated by CHWs were consecutively enrolled into the study after their caregivers gave written informed consent. The children were selected from the CHWs' register and visited at home on day one of treatment and day four after the initiation of treatment.

### Sample size

The sample size was computed based on the formula for comparison of two proportions with adjustment for clustering [23]. We assumed 80% power, 5% level of significance, design effect of two, 81% adherence among children taking AL alone [12], a 20% reduction in adherence among children taking both antimalarials and antibiotics giving adherence of 64.8%, and 10% loss to follow up. Based on these assumptions, a minimum sample size of 258 children in each group was required. We planned to enroll children taking AL in the control arm, AL in the intervention arm, and AL plus amoxicillin in the intervention arm.

### Data collection

The data was collected using a pre-tested questionnaire that was divided into two parts. Part one of the questionnaire collected data on: demographic characteristics of children and caregivers, presenting symptoms of the child, promptness of care seeking, treatment received, and caregiver's perceived severity of illness. Part two of the questionnaire collected data on: understanding of medicines administration instructions, presence of other sick persons in the home, vomiting of drugs, perceived benefit from treatment, and adherence to medicines. Part one of the questionnaire was administered on day one of treatment; patients that had received the first treatment in the evening or at night were interviewed on the next day which was still within the first twenty four hours of treatment. Part two was administered on day four but for patients that had initiated treatment in the evening or at night, they could be interviewed in the afternoon of day four or morning of day five (which would still be within the twenty four hours constituting their day four). The interviews were conducted in Lusoga, the main local language spoken in the area. Children's temperatures as well as their respiratory rates were measured both on day one (baseline) and day four. The medicines packets were also checked, when available, for remaining tablets on day four. The day four visit was unannounced in order to avoid the influence of the impending visit on adherence and to avoid pill dumping that may occur when patients expect their medicines to be checked. Unannounced pill counts give a better representation of adherence than when patients expect a visit from the health provider [24], [25].

### Definitions of variables

The level of adherence was determined as the percentage of prescribed pills that the child had taken correctly over a three day period. The computation was based on caregivers' reports of how the medicines had been administered validated by checking of the medicines' packet to determine if there were any pills and the number of pills that were still present on the day of evaluation (pill counts). If adherence measured by caregiver reports was contradicted by the pill counts, the adherence level obtained from pill counts would be taken. The computed level of adherence was then categorized into: adherent (those that had taken all their prescribed drugs appropriately) and non-adherent (those that had not taken some of their prescribed drugs or had taken them inappropriately). In addition, caregivers were asked if the children had vomited within thirty minutes of taking the medicines and whether they had re-administered these vomited doses. Children that had been classified as adherent were considered as non-adherent if they had not re-administered vomited doses [26].

We further evaluated adherence based on pill counts available in the medicines packet, the caregiver's report of how they had administered the medicines, and what the medicines had been administered with or what foods were taken before the medicines. Based on this we categorized the participants as having: optimal adherence if they had taken all their medicines as prescribed and had taken them with a fatty meal; good adherence if they had taken all their medicines as prescribed but had either not taken the medicines consistently with a fatty meal or had not taken the medicines with a fatty meal at all; and non-adherence if some of the medicines were not taken or were not taken in the correct schedule [27].

Promptness of treatment was defined as seeking care on the day of presentation of symptoms or the next day [28].

### Data management and analysis

The data was double entered into FoxPro and exported to STATA 10 (StataCorp., College Station, TX, USA) for statistical analysis. Descriptive statistics were used to summarize the data and comparisons were made using Analysis of variance or Kruskal Wallis tests for continuous variables or chi-squared or Fishers's exact tests for categorical variables. Pairwise comparisons between the groups following ANOVA or Kruskal Wallis tests were conducted using t-tests or Mann Whitney U tests whose alpha levels were adjusted for multiple comparisons using the Bonferroni correction. Logistic regression was used to determine the factors associated with non-adherence. Unadjusted and adjusted analyses were done. Factors that had p-values less than 0.2 at unadjusted analysis were included in the adjusted analyses together with the treatment group. All analyses were adjusted for similarity between children treated by the same CHW using survey methods (“svy” commands). In addition, agreement between pill counts and caregiver reports in estimation of non-adherence was measured by the kappa statistic.

## Results

A total of 1256 children were enrolled into the study (667 from control arm (control arm), 323 from intervention arm taking antimalarials only (intervention arm AM), and 266 from the intervention arm taking antimalarials and antibiotics (intervention arm AM+AB).

### Demographic and clinical characteristics of the participants

About half of the children in all three groups were female and their median age was 30 months. The caregivers were mostly females, married, living in rural areas, with a mean age of 30 years. The majority had attained primary level education. A higher proportion of children in the intervention arm AM+AB group reported symptoms of cough and fast breathing; had temperature above 37.5°C and fast breathing based on breath counts taken on day one of treatment; lived in urban areas; and had more male caregivers compared to the intervention AM group and the control arm. Demographic and clinical characteristics are summarized in Table 1.

**Table 1:** Demographic and clinical characteristics of 1256 children and caregivers in Iganga-Mayuge DSS.

Characteristic	Sub-category	Control arm	Intervention arm		P-value
		AM (n = 667)	AM (n = 323)	AM+AB (n = 266)	
Sex of child, n (%)	Female	336 (50.4)	181 (56.0)	139 (52.3)	0.25
Age of child in months	Median (min, max#)	31.0 (4.0, 59.0)	27.0 (4.0, 59.0)	31.5 (5.0, 59.0)	0.06
Sex of caregiver, n (%)	Female	625 (93.7)	306 (94.7)	236 (88.7)	0.01
Age of caregiver in years	mean (SD)	30.7 (8.7)	30.7 (9.4)	31.2 (9.3)	0.60
Caregiver education level, n (%)*	None	69 (10.4)	36 (11.1)	23 (8.7)	0.002
	Primary	450 (67.7)	199 (61.0)	176 (66.4)	
	Secondary	130 (19.6)	90 (27.9)	64 (24.1)	
	Tertiary	16 (2.4)	0 (0)	2 (0.8)	
Religion, n (%)	Catholic	65 (9.8)	11 (3.4)	19 (7.2)	0.002
	Protestant	224 (33.9)	125 (38.7)	108 (40.7)	
	Muslim	334 (50.2)	176 (54.5)	127 (47.9)	
	Other	42 (6.3)	11 (3.4)	11 (4.2)	
Marital status, n (%)	Married	592 (88.8)	293 (91.1)	250 (94.0)	0.18
	Single	27 (4.1)	15 (4.7)	6 (2.3)	
	Divorced	37 (5.6)	12 (3.7)	7 (2.7)	
	Widowed	1 (1.7)	2 (0.6)	3 (1.1)	
Residence, n (%)	Rural	600 (90.0)	291 (90.1)	191 (71.8)	<0.001
History of fever, n (%)		647 (97.0)	314 (97.2)	259 (97.4)	0.95
History of cough, n (%)		541 (81.1)	237 (73.4)	247 (92.9)	<0.001
History of fast breathing, n (%)		199 (29.8)	67 (20.7)	109 (41.0)	<0.001
Temperature ≥37.5°C, n (%)		95 (14.2)	34 (10.5)	49 (18.4)	0.02
Fast breathing (measured), n (%)		77 (11.5)	23 (7.1)	94 (35.3)	<0.001

AM – AntimalarialsAB – Antibiotics

# Minimum, maximum.

* Three (3) missing values.

### Comparison of caregiver reports and pill counts in assessing adherence

The agreement (kappa) between caregiver reports and pill counts in classifying children as non-adherent was 0.91. About 7.6% of the children classified as non-adherent by pill counts were classified as adherent by caregiver reports while 0.9% of those classified as adherent by pill counts were classified as non-adherent by caregiver reports.

### Adherence to medicines

The mean adherence to medicines was more than 95% in all three treatment groups but was significantly lower in the control arm (mean 96%) compared to the group in the intervention arm using AL only (mean 99%) and that using both AL and amoxicillin (mean 99%) (p<0.001) (Table 2). There was no difference in adherence between children taking AL alone and children taking AL plus amoxicillin in the intervention arm (p = 0.19). The proportion of children with optimal adherence to medicines was 18% in the control arm, 26% in the intervention arm taking antimalarials only and 23% in the intervention arm taking both antimalarials and antibiotics. Most patients, although taking medicines as prescribed, did not take artemether-lumefantrine with a fatty meal as recommended by the manufacturer for optimal absorption. The proportion of children that took their medicines with a fatty drink or food was only slightly above 25% in all three treatment groups for the three days of taking medicines.

**Table 2:** Adherence to medicines for 1256 children in Iganga-Mayuge DSS.

Characteristic	Control arm	Intervention arm		P-value
	AM(N = 667)	AM (N = 323)	AM+AB (N = 266)	
**Mean overall adherence (SD)**	95.5 (13.4)	99.2 (4.5)	98.5 (6.5)	0.02
Control * *vs* Intervention AM	95.5 (13.4)	99.2 (4.5)		<0.001
Control * *vs* Intervention AM+AB#	95.5 (13.4)		98.5 (6.5)	0.001
Intervention AM+ *vs* Intervention AM+AB #		99.2 (4.5)	98.5 (6.5)	0.19
**Overall non-adherence, n (%)**	92 (13.8)	13 (4.0)	17 (6.4)	<0.001
Overall adherence adjusted for fatty foods taken **, n (%)				
Optimal adherence	118 (17.8)	82 (25.5)	60 (22.6)	0.002
Good adherence	454 (68.5)	227 (70.5)	188 (70.9)	
Non-adherence	92 (13.8)	13 (4.0)	17 (6.4)	
**Overall adherence adjusted for fatty foods taken and vomiting of medicines, n (%)**				
Optimal adherence	112 (16.9)	80 (24.8)	60 (22.6)	<0.001
Good adherence	441 (66.5)	225 (69.9)	185 (69.8)	
Non-adherence	110 (16.6)	17 (5.3)	20 (7.6)	

AM – AntimalarialsAB – Antibiotics.

* taking antimalarials only in control arm.

+ taking antimalarials only in the intervention arm.

# taking combination of antimalarials and antibiotics in the intervention arm.

** missing values (5 caregivers (3 control arm, 1 intervention AM, 1 intervention AM+AB) could not remember what the medicines had been administered with).

Furthermore, when adjustments were made in adherence depending on whether patients that vomited their medicines within thirty minutes had another dose given, the level of non-adherence increased from 14% to 17% in the control arm, from 4% to 5% in the intervention arm taking antimalarials only, and from 6% to 8% in the intervention arm taking antimalarials and antibiotics. About 2% (n = 26) of children that would have otherwise been adherent were not because they vomited within 30 minutes and did not have another dose administered. Many children that vomited within thirty minutes of taking the medicines did not have another dose re-administered (61%, 25/41 in the control arm; 50%, 4/8 in the intervention arm taking antimalarials only; and 40%, 4/10 in the intervention arm taking antimalarials and antibiotics, p = 0.49).

The most cited reasons for non-adherence were forgetfulness to give the medicines (38%, n = 24), caregiver's perception of improvement or recovery in the child (14%, n = 9), and vomiting (13%, n = 8) (Table 3).

**Table 3:** Reasons for missing medicines among the 61 children who did not adhere to the prescribed treatment, Iganga-Mayuge DSS.

Item	Frequency	Percent
I forgot to give the medicine	24	38.1
Child improved/recovered	9	14.3
The child was vomiting	8	12.7
There were too many tablets	7	11.1
I did not have food or drink to give the child	3	4.8
Did not understand the instructions well	3	4.8
Other*	7	11.1

* Includes being away, child experiencing adverse reaction, had other drugs to take.

### Factors associated with non-adherence to medicines

At adjusted analysis, the association between the treatment received and non-adherence was not statistically significant. However, no reported fever (OR = 3.3, 95%CI = 1.6–6.9), seeking care after two or more days (OR = 2.2, 95%CI = 1.3–3.7), not understanding instructions given (OR = 24.5, 95%CI = 2.7–224.5), vomiting (OR = 2.6, 95%CI = 1.2–5.5), and caregivers' perception that the child's illness was not severe (OR = 2.0, 95%CI = 1.1–3.8) were associated with non-adherence. Factors associated with non-adherence at unadjusted and adjusted analysis are summarized in Table 4.

**Table 4:** Factors associated with non-adherence at unadjusted and adjusted analyses in Iganga-Mayuge DSS.

Characteristic	Sub-category	Unadjusted OR (95%CI)	P-value	Adjusted OR (95%CI)	P-value
Treatment group	Intervention – AM+AB	1.0		1.0	
	Intervention – AM only	0.6 (0.3–1.3)	0.22	0.5 (0.2–1.2)	0.16
	Control arm	2.3 (1.2–4.7)	0.02	1.9 (0.9–3.8)	0.07
Child's age	<36 months	1.0			
	≥36 months	1.5 (1.1–2.0)	0.02		
Had fever	Yes	1.0		1.0	
	No	3.3 (1.6–6.9)	0.002	3.2 (1.5–6.8)	0.003
Difficult breathing	Yes	1.5 (0.9–2.5)	0.11		
	No	1.0			
Time before care seeking	One day or less	1.0		1.0	
	Two days or more	2.6 (1.5–4.4)	0.001	2.2 (1.3–3.7)	0.003
Gave other treatment before CMD	Yes	1.9 (1.3–2.8)	0.002		
	No	1.0			
Understood instructions	Yes	1.0		1.0	
	No	39.1 (4.0–387.5)	0.002	24.5 (2.7–224.5)	0.005
Breathing rate - day 1		0.97 (0.95–1.01)	0.10		
Vomiting	Yes	2.8 (1.4–5.3)	0.003	2.6 (1.2–5.5)	0.02
	No	1.0		1.0	
Rural residence	Urban	1.0			
	Rural	2.4 (1.3–4.7)	0.008		
Education of caregiver	Primary and below	1.9 (0.9–3.8)			
	Post primary	1.0	0.07		
Perceived illness severity	Very/moderate	1.0		1.0	
	Not severe	2.1 (1.1–4.0)	0.03	2.0 (1.1–3.8)	0.04

AM – AntimalarialsAB – Antibiotics.

## Discussion

We found high adherence to both antimalarials and antibiotics given by CHWs. Adherence to the combination of antimalarials and antibiotics was not significantly different from adherence to antimalarials alone. Non-adherence was associated with: no history of fever, seeking care after two days of illness or more, not understanding the medicines administration instructions given by the CHW, vomiting for the current illness episode and caregivers perceiving the illness not to be severe.

Our findings show no significant difference in adherence between children who took AL alone and those who took AL and amoxicillin. This is in contrast to our hypothesis that increased pill burden might lower adherence. Increased pill burden has been reported as a hindrance to adherence [4]. A possible explanation for our findings is that children who were treated with combinations of AL and amoxicillin may have had symptoms like fast or difficult breathing which their caregivers may perceive to be severe [29] and are therefore motivated to administer the medicines hence adherence to treatment.

The levels of adherence to artemether-lumefantrine found in this study are similar to the adherence levels of 71–97% that were reported in studies done in Ghana, Uganda and Nigeria [10], [11], [12] among children treated by CHWs. They are also similar to adherence levels to AL among children treated at health facilities (64–95%) in Uganda, Kenya and Tanzania [27], [30], [31], [32], [33]. The high adherence may be due to use of pre-packaged medicines with pictures showing how the medicines should be administered. This has been shown to improve adherence [30]. It is also likely that the caregivers and CHWs have good communication between them because the CHWs live in the same area and speak the same language. This may improve understanding of the medicines administration instructions given by CHWs. However, the adherence levels in our study could have been an overestimate because the exact timing of doses was not considered. Mistiming of doses has been found in some studies as the most common source of non-adherence [18]. In addition, the visit at home on day one of treatment seeking may have influenced adherence because the caregivers were asked about the medicines the children had received from the CHW. However, efforts were made to minimize the effects of home visits on adherence by not informing the caregiver of the day four visit.

Although a high level of adherence was observed, the medicines were not taken with a fatty meal as has been recommended to improve the absorption of AL [34]. This resulted in low levels of “optimal” adherence (18% in control arm, 26% in the intervention arm taking antimalarials only, and 23% in the intervention arm taking combination of antimalarials and antibiotics). Caregivers may not be able to give fatty foods when administering artemether-lumefantrine either because of the child's illness or because they lack the fatty foods. The feasibility of this recommendation needs to be evaluated further and the instructions given to caregivers should identify the easily accessible fatty foods in each setting. The level of optimal adherence found in our study is lower than the 34% reported in a health facility setting in Uganda [27]. In that study, all the caregivers were instructed to give medicines with a fatty food.

As in previous studies, forgetfulness was cited as a reason for non-adherence to treatment [26], [35] highlighting the need to devise strategies for reminding caregivers about the medicines administration schedule. Other reasons given for missing medicines including recovery and vomiting have also been cited in other studies [26]. Vomiting was also significantly associated with non-adherence at adjusted analysis. The citing of recovery or improvement of the child as a reason for missing medicines points to a need for better counseling of caregivers to ensure that children continue to receive their medicines even when they seem to improve. Caregivers should also be advised on what to do in case the child vomits, and encouraged to collect more drugs from the CHWs to replace the vomited doses. About half of the caregivers whose children vomited within thirty minutes of taking the medicines did not re-administer vomited doses. These findings are slightly higher than in a study in Ethiopia where only five of 14 children that vomited had medicines re-administered [18].

At unadjusted analysis, the children in the control arm had lower adherence than those in the intervention arm either taking antimalarials alone or taking combinations of antimalarials and antibiotics. This difference was not apparent at adjusted analysis. The lower adherence seen in the control arm may have been due to confounding relationships with understanding medicines administration instructions and promptness of care seeking. The proportion of children that did not understand medicines administration instructions was higher in the control arm and this may have contributed to the lower adherence seen. In addition, a lower proportion of children in the control arm sought care promptly compared to the intervention arm with antimalarials plus antibiotics. This may have been a reflection of perception of the illness not to be severe leading to delayed care seeking. Perception of illness not to be severe may lower adherence as found in this study. The CHWs in both the intervention and control arm had the same frequency of contact with trainers and supervisors and therefore would not be expected to have differences in the instructions they give to caregivers.

Children that did not report a history of fever were more likely to be non-adherent. This may be due to perception of severity of illness. A previous study reported that caregivers were more likely to seek care for children with fever mainly due to perception of illness as severe [36], while another study reported inappropriate medicines use in children without fever [21]. In further support of this argument, caregivers that perceived their child's illness not to be severe were more likely to be non-adherent. This finding is in agreement with what has been reported in the literature that perceptions of illness severity influence medication use [37]. It is surprising that children without a history of fever were treated with AL. A possible explanation for this is that the caregivers may not have detected the fever but the CHW detected it when the child was taken for treatment and therefore gave AL.

Children that sought treatment promptly were more likely to be adherent. This may be due to perception of severity of illness. The caregivers may delay to seek care because they do not perceive the illness as severe and perception of severity of illness was associated with adherence in the current study. The finding is similar to that in studies in Kenya and Ethiopia where patients that had waited for more than twenty four hours to seek care had lower adherence [18], [32].

Although only a few caregivers reported not having understood the instructions given by the CHW, this factor was significantly associated with non-adherence. In addition, a few caregivers (4.8%) cited not understanding the medicines administration instructions as the reason why they missed medicines. Another study done in Zanzibar found that 53% of the caregivers that did not adhere to treatment did so because they could not remember or had misunderstood the instructions received [26]. This finding suggests the need to ensure that all caregivers understand the medicines administration instructions. This result should however be interpreted with caution because of the imprecision due to the small numbers of caregivers reporting not understanding instructions.

### Methodological issues

The use of self reports of how patients take medicines has been associated with problems of recall and overestimation of adherence [4]. The problem of recall may have been minimized by the short period over which patients were required to recall since adherence was measured on day four since presentation to the CHW. In addition, we tried to minimize the overestimation of adherence by combining self reports with unannounced home pill counts which have been shown to improve adherence assessment and reduce the problem of pill dumping that may occur when patients are asked to present their medicines packets for assessment [24]. Nevertheless, it is possible that patients may not have taken all the medicines that are removed from the packet [4]or they may not have taken them as they reported since the dosing schedule assessment still depended on the caregiver report. We also did not assess the exact timing of the doses and this could have led to underestimation of non-adherence.

## Conclusions

The treatment of children by CHWs with combinations of antimalarials and antibiotics did not lower adherence compared to antimalarials alone. The children achieved high levels of adherence in both situations. However, more emphasis needs to be laid on counseling caregivers to ensure that they understand the dosing regimens. In addition, caregivers need to be advised to continue with the medicines even when the child seems to improve and to re-administer doses that have been vomited. The CHWs should advise the caregivers to collect additional drugs in cases where some doses have been vomited.
